# Role of somatostatin receptor 2 (SSTR2) in pituitary adenomas

**DOI:** 10.1002/ctm2.70435

**Published:** 2025-08-06

**Authors:** Yvonne Power, Jacklyn Liu, Umar Rehman, Adelina Kjerimi, Volker Schartinger, Nicholas Counsell, Joachim Starup‐Hansen, Nicola Newall, Valerie J. Lund, Ahmed Mohyeldin, Juan C. Fernandez‐Miranda, Jonathan Lavezo, Zara M. Patel, Jayakar V. Nayak, Peter H. Hwang, Sebastian Brandner, Hani Marcus, Robert B. West, Matt Lechner

**Affiliations:** ^1^ Division of Surgery and Interventional Science University College London London UK; ^2^ UCL Cancer Institute University College London London UK; ^3^ UCL Queen Square Institute of Neurology London UK; ^4^ Department of Otorhinolaryngology Medical University of Innsbruck Innsbruck Austria; ^5^ Cancer Research UK and UCL Cancer Trials Centre University College London London UK; ^6^ Wellcome/EPSRC Centre for Interventional and Surgical Sciences Charles Bell House University College London London UK; ^7^ Division of Neurosurgery National Hospital for Neurology and Neurosurgery, Queen Square London UK; ^8^ University College London London UK; ^9^ Department of Neurosurgery University of California‐Irvine Irvine California USA; ^10^ Department of Neurosurgery Stanford University School of Medicine Palo Alto California USA; ^11^ Department of Neuropathology University Medical Center of El Paso El Paso Texas USA; ^12^ Division of Rhinology and Endoscopic Skull Base Surgery Department of Otolaryngology—Head and Neck Surgery Stanford University School of Medicine Palo Alto California USA; ^13^ Division of Neuropathology UCL Queen Square Institute of Neurology London UK; ^14^ Department of Pathology Stanford University School of Medicine Palo Alto California USA

1

Dear Editor,

In this study, we present a large evaluation of somatostatin receptor 2 (SSTR2) expression in pituitary adenomas (PA), identifying thyrotroph and somatotroph lineages as those with the most frequent and intense expression. These findings provide important insights and contribute to the groundwork for further exploration of SSTR2's diagnostic utility and the therapeutic potential of somatostatin (SST) analogues and other SSTR2‐targeting approaches across a variety of PAs.

PAs are typically benign, slow‐growing tumours originating from the anterior pituitary gland. Although the term ‘pituitary neuroendocrine tumour’ (PitNET) has been proposed to reflect their neuroendocrine nature, the Pituitary Society supports retaining the term ‘pituitary adenoma’, arguing that the new terminology may cause unnecessary patient anxiety without improving clinical care. Consequently, ‘pituitary adenoma’ remains the widely used term in clinical practice.^1^, ^2^ PAs are the second most common type of intracranial neoplasm.^2^ They are categorised by lineage‐specific transcription factors: T‐box transcription factor 1 (TPIT), corticotroph adenomas (ACTH‐secreting), steroidogenic factor‐1 (SF1), gonadotroph adenomas (FSH and LH‐secreting) and pituitary transcription factor 1 (PIT1) lineage adenomas, including somatotroph (GH‐secreting), lactotroph (PRL‐secreting), mammosomatotroph and thyrotroph tumours.^2^

SSTR2 is a G‐protein‐coupled receptor, which is highly expressed in neuroendocrine and some head and neck tumours and serves as a potential biomarker and therapeutic target.^3^, ^4^, ^5^ Its natural ligand, SST, has antiproliferative, antisecretory and pro‐apoptotic effects.^5^ SST analogues (SSAs) have shown effectiveness in treating SSTR2‐expressing neuroendocrine tumours.^6^ First‐generation SSAs, octreotide (OCT) and lanreotide (LAN), are approved for the treatment of PAs.^3^ SSTR2 expression has been observed in PAs, with lack of expression linked to octreotide resistance, potentially impacting treatment outcomes.^7^, ^8^

In this retrospective cohort from two sites (University College London/University College London Hospital, UK, and Stanford University, USA), we evaluated SSTR2 expression in 310 PA samples using a validated immunohistochemical protocol.^3^, ^4^ Assessment of SSTR2 expression was performed using tissue microarrays (TMAs) constructed from formalin‐fixed paraffin‐embedded specimens collected from these two sites. Tumour areas were marked by two expert histopathologists, and three  0.6 mm cores were punched from these areas and arrayed into a recipient TMA donor block to account for tumour heterogeneity.

Immunohistochemical staining was conducted using a standardised protocol shared between both centres, employing the same rabbit monoclonal anti‐SSTR2 (UMB1) antibody (ab134152, Abcam) and detection system (ultraView Universal DAB Detection Kit, Ventana). Evaluation of staining intensity and extent was performed independently by blinded expert pathologists at each institution, using a validated scoring system with defined categories for intensity (0 = negative to 3 = strong) and extent (i.e., proportion of positive cells) of staining (0 = 0% to 4 = 81%‒100%) (Supporting Information).^3^ Although the review was not centralised, the use of a validated standardised quantification method between sites ensured comparable analysis (Figure 1).

**FIGURE 1:**
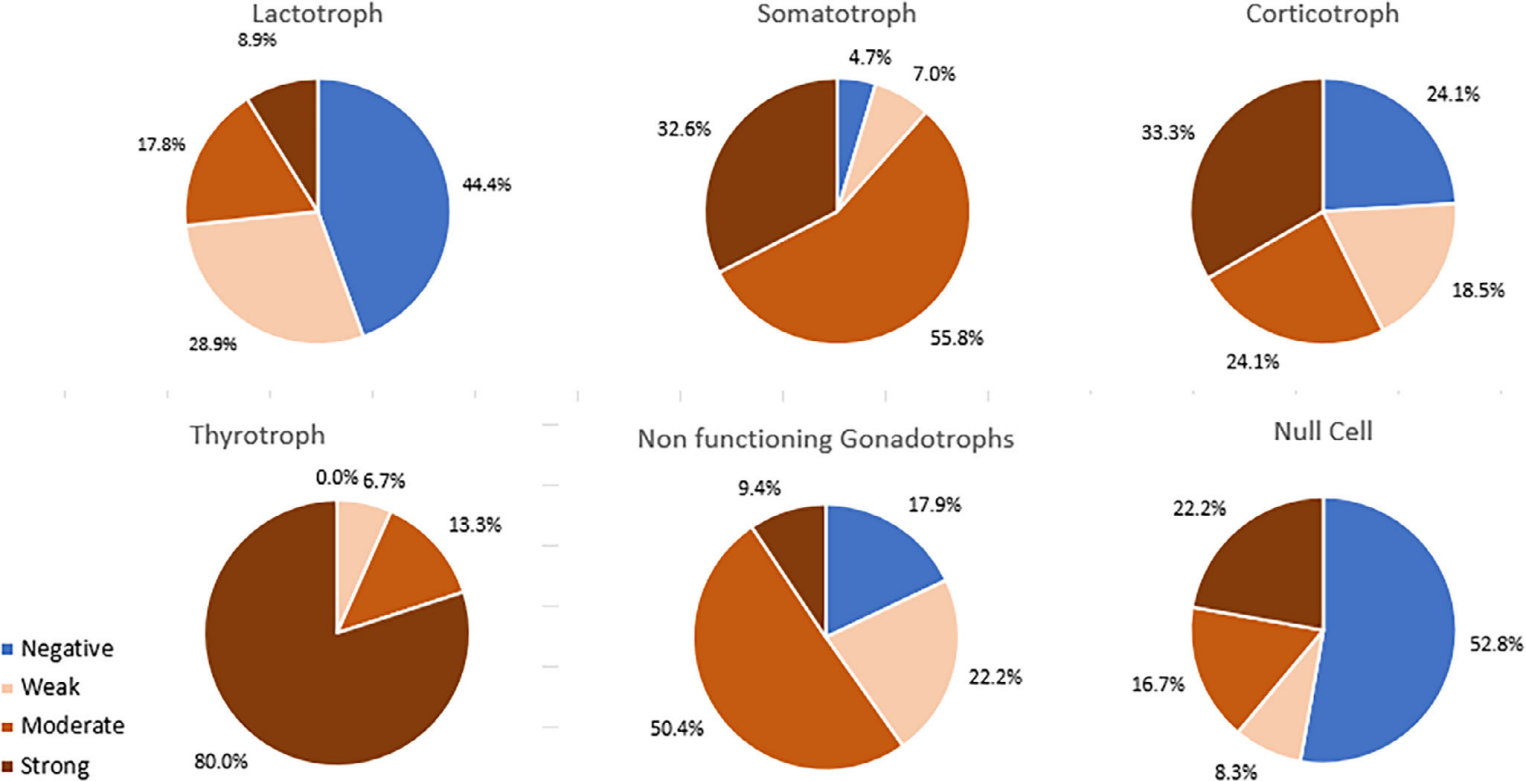
Representative pie chart of staining intensities (negative, weak, moderate, strong) for each tumour lineage. For tumours with strong/moderate expression. Staining intensity refers to the strength of the staining signal is the cells found to be positive for somatostatin receptor 2 (SSTR2).

The cohort included 43 somatotroph, 54 corticotroph, 15 thyrotroph, 45 lactotroph, 36 null‐cell and 117 gonadotroph adenomas. The results of the immunohistochemical analysis are summarised in Tables 1 and S1. Thyrotroph and somatotroph adenomas exhibited the highest proportions of positively stained cells.

**TABLE 1:** Characteristics of patients with pituitary adenoma.

	Total (*n* = 310)
Tumour lineage (*n* = 310)	
Total lactotrophs (%)	45 (14.5)
Total somatotrophs (%)	43 (13.9)
Total corticotrophs (%)	54 (17.4)
Total thyrotrophs (%)	15 (4.8)
Total gonadotrophs (%)	117 (37.7)
Total null cell (%)	36 (11.6)
Age (*n* = 208)	
Mean (SD)	54 (16.1)
Median (range)	51 (67)
Sex (*n* = 208)	
Female	126
Male	82
Tumour size (*n* = 175)	
Micro (%)	33 (18.9)
Macro (%)	140 (80)
Giant (%)	2 (1.1)

To place our findings in context, a systematic review of the literature was conducted, which yielded 46 articles including a large study conducted by Fuchs et al. of 299 PAs (Supporting Information refs. 4‒49). Compared to Fuchs et al., our findings showed consistently higher SSTR2 positivity across all tumour lineages: thyrotrophs (100% vs. 18%), somatotrophs (95.3% vs. 64%), gonadotrophs (82.8% vs. 15%), corticotrophs (75.9% vs. 28%), lactotrophs (55.6% vs. 22%) and null cell adenomas (47.2% vs. 22%).^9^ These differences may be attributed to geographical differences and variations in staining and scoring methodology. Our results align with prior reports demonstrating SSTR2 positivity in 89%‒100% of thyrotrophs, 50%‒100% of somatotrophs, and 0%‒90% of gonadotrophs, although earlier studies are limited by small sample sizes and methodological inconsistencies (Supporting Information refs. 4‒49).

To compare staining intensity across tumour lineages in the present cohort, logistic regression was undertaken using lactotrophs as the reference lineage (Table S2). Compared to lactotrophs, each of thyrotrophs, somatotrophs, NF gonadotrophs and corticotrophs demonstrated increased odds of SSTR2 positivity. Similar results were observed for staining extent (Table S3). We further explored the relationship between clinicopathological factors (age, sex, tumour size) and the extent and intensity of SSTR2 expression. There was no evidence of an association between each of age and sex and SSTR2 expression. For associations with tumour size, macro‐tumours were used as the reference due to their prevalence. Micro‐tumours demonstrated odds ratios of 1.386 (95% confidence interval [CI]:  0.695‒2.763; *p* = 0.355) for intensity and  0.720 (95% CI:  0.364‒1.439; *p* = 0.346) for extent, although none of these were statistically significant.

Multivariable regression analysis was then undertaken to determine the variations in staining extent and intensity across the lineages while accounting for age, sex and tumour size in 173 cases with complete clinical data (Tables S4 and S5). There were no statistically significant differences in staining percentage regarding age, sex and tumour size. However, when compared to the reference group (lactotroph tumours), all other tumour lineages exhibited greater odds of SSTR2 expression.

Overall, in this study, thyrotrophs displayed the highest SSTR2 expression, with 100% positivity and 80% demonstrating strong staining. This suggests SSTR2 may serve as a useful marker for radionuclide imaging, aiding diagnosis, treatment planning and monitoring response to therapies such as Octreotide. Low SSTR2 expression is associated with resistance to SSAs, and a low SSTR2/SSTR5 ratio also predicts poor therapeutic response. Although the interaction between SSTR2 and SSTR5 was beyond the scope of this study, future research using larger datasets should investigate their combined role in treatment response. At this stage, most clinically approved therapeutic interventions target SSTR2 and our study highlights the potential candidacy of standardised SSTR2 assessment as a companion diagnostic biomarker in PAs.

Some limitations of the study should be acknowledged. First, its retrospective design limit the clinical outcome data that are available, which hinders robust statistical analysis. Second, the absence of SSTR5 assessment restricts a comprehensive understanding of receptor involvement. Nonetheless, the study's large sample size and use of a standardised and validated scoring methodology remain key strengths. Future prospective studies should evaluate the predictive role of both SSTR2 and SSTR5 expression in treatment response and compare against clinical outcome metrics.

In conclusion, this study evaluates SSTR2 expression in a large dataset of PA, highlighting its potential as a diagnostic biomarker, particularly in thyrotroph and somatotroph lineages, where expression was highest. These findings offer insight into which lineage may respond better to pharmacological treatments and how differential expression may guide therapeutic decision‐making.

## AUTHOR CONTRIBUTIONS

*Conceptualisation*: Yvonne Power, Jacklyn Liu, Sebastian Brandner, Hani Marcus, Robert B. West and Matt Lechner. *Methodology*: Yvonne Power, Volker Schartinger, Jacklyn Liu and Matt Lechner. *Data acquisition*: Sebastian Brandner, Joachim Starup‐Hansen, Nicola Newall, Robert B. West and Matt Lechner. *Formal analysis and investigation*: Yvonne Power, Jacklyn Liu, Adelina Kjerimi, Umar Rehman Volker Schartinger, Robert B. West and Matt Lechner. *Writing—original draft preparation*: Yvonne Power, Jacklyn Liu, Nicholas Counsell, Umar Rehman, Robert B. West and Matt Lechner. *Supervision*: Sebastian Brandner, Hani Marcus, Robert B. West and Matt Lechner.

## CONFLICT OF INTEREST STATEMENT

The authors declare they have no conflicts of interest.

## ETHICS STATEMENT

This retrospective study involving clinical data and tissue specimens from human participants was conducted in accordance with the ethical standards of the institutional and national research committees, and with the 1964 Helsinki Declaration and its later amendments or comparable ethical standards. Ethical approval was obtained from all participating institutions, including the BRAIN UK, South Central–Hampshire B Research Ethics Committee (Ref. No. 23/006), the Stanford IRB Committee (IRB‐43567), and the University College London Research Ethics Committee (UCL REC No. 9609/002; ML/VJL), which also granted approval for multi‐center data analysis.

## Supporting information



Supporting Information

## Data Availability

The data that support the findings of this study are available from the corresponding author upon reasonable request.
