# Supplementary material for: Role of somatostatin receptor 2 (SSTR2) in pituitary adenomas
Source: Clin Transl Med. 2025 Aug 6;15(8):e70435. doi: 10.1002/ctm2.70435 (PMC12328241; doi:10.1002/ctm2.70435)
Supplement: Supplementary file 1 — Supporting Information [file CTM2-15-e70435-s001.docx]

**Supplemental Information**

**Supplemental Materials and Methods**

***Systematic Review***

A systematic review was performed on the available, published literature pertaining to the expression of SSTR2 in PA including corticotroph, somatotroph, lactotroph, thyrotroph and gonadotroph adenomas and all NFPAs. The review was carried out on PubMed, EMBASE, DARE, Dynamed and Scopus including all articles from 1990-2023. The search strategy is seen in Supplemental Table 6.

***Materials***

All resected pituitary tumour specimens archived in the pathology departments of the participating institutions were reviewed for eligibility. Accordingly, all adult patients (aged 18 years or older) with a confirmed pituitary tumour who had undergone surgical excision were included.

Formalin-fixed paraffin-embedded (FFPE) specimens from patients diagnosed with PA were sectioned and obtained from University College London/University College London Hospital, UK and from Stanford University, USA. Ethical approval was obtained from each institution (Stanford University Research Ethics Committee, ref. IRB-43567; BRAIN UK, South Central-Hampshire B Research Ethics Committee, ref. no. 23/006), with further ethical approval for multicentre data analysis from University College London Research Ethics Committee (UCL Research Ethics Committee, ref. no. 9609/002; ML/VJL). Inclusion criteria required a confirmed histopathological diagnosis of PA or any of the lineages using WHO 2017 transcription factor staining tumour lineage. Histological characterisation and sample selection was performed by neuropathologists from different centres, highly experienced in the evaluation of PAs [1].

***Tissue Microarray Construction***

Tissue microarrays (TMA) were constructed from 102 specimens from Stanford University Hospital, Stanford, USA. Tumour areas were marked by an expert pathologist and TMAs constructed using a TMA Grand Master instrument (3D HISTECH, Budapest, Hungary). Three cores (0.6 mm) were punched from the marked tumour areas and arrayed into a recipient TMA donor block. Sections from individual blocks were used for staining of UCL/BRAIN UK cases.

***SSTR2 Protein Immunohistochemistry***

A standardised staining protocol was used to perform the immunohistochemistry in both institutions, using the Roche Ventana BenchMark ULTRA (Ventana Medical systems, Tuscon, AZ, USA), and shared between centres by the lead team. SSTR2 was detected using a rabbit monoclonal antibody UMB1 (UMB1 clone, ab134152) (Abcam, Cambridge, UK), with the ultraView Universal DAB Detection Kit from Ventana, as previously described by *Lechner et al* [2].

Staining results were evaluated by blinded expert pathologists experienced in the scoring of SSTR2. The tissue sections were dichotomously scored as being positive or negative, based on the intensity of staining. This was scored as 4 categories (0: negative, 1: weak staining, 2: moderate staining, 3: strong staining) (Supplemental Figure 1). The extent of staining was scored as 5 categories (0=0%; 1= <30%; 2=31-60%; 3=61-80%; 4=81-100%) [2]. Well-characterized olfactory neuroblastoma specimen, as previously described by Lechner et al, were used as positive and negative controls [2,3]. SSTR2 expression was assessed using the semi-quantitative Quick Score method, as originally described by Detre et al. This system combines the proportion of positively stained tumour cells with staining intensity, generating a composite score ranging from 0 to 18 [4].

Cases classified as “null cell” adenomas underwent comprehensive immunohistochemical evaluation. Staining for lineage-defining transcription factors (Pit-1, T-Pit, and SF-1) and GATA3 was performed and found to be negative in all such cases. GATA3 immunostaining was complete for the cohort, as verified using the TMAD platform. While electron microscopy was not performed, CAM5.2 (cytokeratin) staining was carried out and supported the null cell classification by demonstrating an absence of lineage-specific cytokeratin patterns. Classification was confirmed through consensus between neuropathologists, and the designation of “null cell” adenoma was based on confirmed negativity for both hormones and transcription factors.

***Statistical Methods***

Statistical analysis was performed using IBM SPSS Statistics for Windows version 29.0. The evaluation of SSTR2 expression across different lineagesemployed an ordinal logistic regression model, with a significance threshold set at p < 0.05. A univariable ordinal logistic regression model was employed to determine the association between tumour lineages and staining intensity and staining percentage. Furthermore, a similar analysis was conducted to investigate the association between age, gender, tumour size, and SSTR2 expression. A multivariable regression model was performed to determine the combined influence of these variables on the protein expression.

**Supplemental Results**

***Systematic Review***

Data was collected and findings were recorded based on 46 published articles [5-49], which are summarised in the PRISMA flow diagram in Supplemental Figure 2.

Twenty-two studies examined the expression of SSTR2 protein in somatotroph adenomas using immunohistochemistry (IHC). SSTR2 expression was observed in the large majority of cases (80% to 100%) with strong staining intensity in 39% to 84% of cases and immunoreactivity scores (IRS) between 4 and 12 [5-19].

Several studies have explored the relationship between SSTR2 expression and response to Somatostatin Receptor Ligand (SRL) treatment. Herkenhoff et al observed a median IRS of 6 in 52 somatotroph adenomas evaluated. 31 of these patients were treated with SRLs with SSTR2 found to be higher in responsive patients compared to non-responsive patients, although this difference was not statistically significant [20]. Chiloiro et al investigated 6 cases that had complete resistance to conventional SRLs. The authors reported SSTR2 expression in two cases and found that SSTR2 expression was higher in control cases, which were responsive to conventional SRLs [21].

Seven studies evaluated the SSTR2 expression between densely granulated (DG) and sparsely granulated (SG) somatotrophs. Chinezu et al, reported that 93% of all tumours were positive for SSTR2 with 72% of DG tumours demonstrating high expression. The authors also determined the SSTR2 expression in 15 ACTH secreting adenomas (all were negative for SSTR2), 23 FSH/LH secreting adenomas (26% positive), 7 pure PRL secreting adenomas (all negative) and 3 TSH secreting adenomas (all highly expressed SSTR2) [21]. Soukup et al, evaluated 110 pituitary tumours (45 SG somatotrophs adenomas, 29 DG somatotroph adenomas, 26 somatotroph adenomas with PRL expression and 10 adenomas with plurihormonal expression). The SG group had significantly lower expression of SSTR2 compared to the other groups. 31 cases received SRL pre-treatment where higher SSTR2 expression levels were associated with a better response to therapy [23]. Overall, higher expression was seen in the densely granulated tumours compared to the sparsely granulated tumours [24-28].

Franck et al, evaluated SSTR2 expression in 39 GH secreting adenoma cases where patients either underwent surgery alone, surgery with neoadjuvant SRL or surgery with neoadjuvant SRL and pegvisomant; the average IRS for drug naïve and monotherapy groups was 6 whereas the combination group was 2 [29]. Muhammad et al observed SSTR2 expression in 100% of their cohort with higher staining observed in patients who received SRL treatment before surgery (median IRS:12) compared to those who did not (median IRS:8) [30]. Comparatively, IHC was performed on 14 GH secreting adenomas that had no pre-treatment before surgery and 20 tumours where patients had received octreotide prior to surgery. All tumours in the first group were positive for SSTR2 and 65% of group two. The staining intensity was not reported. Patients that did not respond to the treatment had no SSTR2 expression [31]. A similar study demonstrated a median IRS score of 6 where patients who received SRL treatment had lower levels of SSTR2 [49]. Likewise, 55 somatotrophs (14 co-expression with prolactin) were evaluated by Venegas-Moreno et al [33]. 23 tumours demonstrated a staining score of 3+ with the remaining having lower staining patterns. All patients received SRL treatment before surgery [32].

Further studies compared SSTR2 expression between the various PA lineages. A study by Fuchs et al, assessed the SSTR2 expression in 299 PA. SSTR2 expression was observed in 64% of somatotrophs, 28% of corticotrophs, 22% of lactotrophs, 18% of thyrotrophs, 22% of null cell adenomas and 15% of gonadotrophs [34]. Thodou et al observed positive SSTR2 expression in nine TSH secreting adenomas with three displaying strong staining intensity [35]. SSTR2 expression in non-functioning and functioning thyrotrophs were examined by Wang et al. There was positive immunostaining in 89% and 94%, respectively [36]. Regarding TSH-secreting adenomas, strong positive expression has been demonstrated in two studies [37,38]. Regarding corticotrophs, Van der Pas et al observed positive expression in 43.5% of cases (10/23), although the mean IRS was low (1.3 to 2.0) [39]. SSTR2 has also been demonstrated in a case report of a 68-year-old male with corticotroph adenoma [40]. In a separate case report, a 41-year-old woman with a lactotroph adenoma was investigated with 5% of the tumour demonstrating positive staining [41].

Nine studies investigated SSTR2 expression in non-functioning adenomas. 118 gonadotroph tumours were analysed by Lee et al. (25% positive). Interestingly, in ten recurrent tumour samples, the SSTR2 IRS score slightly increased. These results were validated on a further 27 samples [42]. Fusco et al reported expression in 26 non-functioning adenomas with positive expression in 46% of the cohort [43]. Ramirez et al observed SSTR2 staining in 60% of 74 null cell adenomas and gonadotrophs [44]. SSTR2 expression was observed in an additional cohort of non-functioning gonadotrophs by Pisarek et al, who found that recurrent tumours had higher levels of SSTR2 [45]. In their cohort of ten non-functioning adenomas, Gruszka et al observed positive expression in seven cases; the lineages included in the analysis was not stated [46]. Øystese et al, investigated 42 gonadotrophs, six null cell adenomas and six ACTH secreting adenomas. There was no positive staining for the null cell adenomas and the other two tumour types showed very weak staining (IRS 1) [47]. SSTR2 expression was evaluated in nine non-functioning adenomas by Tjörnstrand et al. Four gonadotrophs displayed high expression, three displayed moderate expression, while 2 corticotrophs were negative [48]. Chinezu et al, compared functioning and non-functioning plurihormonal somatotroph tumours; non-functioning tumours had lower SSTR2 straining overall [49]. An older study by Pawlikowski et al, demonstrated positive SSTR2 expression in four of five null cell adenomas tested and eleven of thirteen gonadotrophs, six of which had a strong staining pattern [50].

**Limitations**

This study has limitations. All tissues used were from patients with PA evaluated using a scoring algorithm from our previous work, allowing comparison across tumour types. Moreover, the response to treatment from tumour samples was not available due to availability of follow up data. Furthermore, the literature lacks standardization, with some studies using Immunoreactivity Scoring (IRS) while others classify SSTR2 as positive/negative or weak/strong. This inconsistency makes interpretation challenging. Additionally, other factors may influence SSTR2 expression in PA. For example, He et al. found a correlation between SSTR2 expression in glioma patients and epilepsy syndrome [51]. Treatment history also affects SSTR2 expression [52]. Nonetheless, this is a large dataset on SSTR2 expression in pituitary adenomas. Future work will integrate this dataset with others to enhance understanding. SSTR2:SSTR5 ratio was not evaluated in the present study and future work should incorporate larger data sets of pituitary adenomas and determine the interplay between both receptors across a variety of pituitary adenomas. Previous studies have suggested that a ratio greater than 1.3 might be linked to an improved treatment response [7]. Future research could explore this ratio further to gain a deeper understanding of how interactions between SST subtypes influence intracellular signalling and the efficacy of somatostatin analogues [7]. Clinical outcome data was not available, so no conclusions could be drawn regarding the relationship between SSTR2 expression and adenoma growth, recurrence, or responsiveness to somatostatin analogue treatment. Finally, SSTR2 staining was assessed solely based on intensity and extent, without differentiating between cytoplasmic and membranous localization or evaluating the extent of membranous immunoreactivity.

**Supplemental Tables**

**Supplemental Table 1.** SSTR2 staining intensity and staining percentage across pituitary adenoma lineages.

| **Staining Intensity** | Lactotroph | Somatotroph | Corticotroph | Thyrotroph | NF Gonadotroph | Null Cell |
| --- | --- | --- | --- | --- | --- | --- |
|  | (n=45) | (n=43) | (n=54) | (n=15) | (n=117) | (n=36) |
| 0 | 20(44.4%) | 2(4.7%) | 13(24.1%) | 0(0.0%) | 21(17.9%) | 19(52.8%) |
| 1 | 13(28.9%) | 3(7.0%) | 10(18.5%) | 1(6.7%) | 26(22.2%) | 3(8.3%) |
| 2 | 8(17.8%) | 24(55.8%) | 13(24.1%) | 2(13.3%) | 59(50.4%) | 6(16.7%) |
| 3 | 4(8.9%) | 14(32.6%) | 18(33.3%) | 12(80.0%) | 11(9.4%) | 8(22.2%) |
| **Staining Percentage** | Lactotroph | Somatotroph | Corticotroph | Thyrotroph | NF Gonadotroph | Null Cell |
|  | (n=45) | (n=43) | (n=54) | (n=15) | (n=117) | (n=36) |
| 0 (0%) | 20(44.4%) | 2(4.7%) | 13(24.1%) | 0(0.0%) | 20(17.2%) | 19(52.8%) |
| 1 (<30%) | 13(28.9%) | 6(14.0%) | 14(26.0%) | 2(13.3%) | 28(24.0%) | 4(11.1%) |
| 2 (31-60%) | 5(11.1%) | 12(27.9%) | 10(18.5%) | 0(0.0%) | 21(18.0%) | 3(8.3%) |
| 3 (61-80%) | 0(0.0%) | 8(18.6%) | 4(7.4%) | 3(20.0%) | 14(12.0%) | 2(5.6%) |
| 4 (81-100%) | 7(15.6%) | 15(34.9%) | 13(24.1%) | 10(66.7%) | 33(28.2%) | 8(22.2%) |

**Supplemental Table 2.** Association between pituitary lineages and SSTR2 staining intensity.

| Lineage (Reference = Lactotroph) | Odds Ratio | Sig. | 95% Confidence Interval | |
| --- | --- | --- | --- | --- |
|  |  |  | Lower Bound | Upper Bound |
| Thyrotrophs | 55.761 | 0.000 | 13.843 | 224.612 |
| Corticotrophs | 4.166 | 0.000 | 1.988 | 8.730 |
| Somatotrophs | 9.111 | 0.000 | 4.074 | 20.373 |
| Gonadotrophs | 2.898 | 0.001 | 1.528 | 5.497 |
| Null | 1.196 | 0.664 | 0.534 | 2.681 |

**Supplemental Table 3.** Association between pituitary lineages and SSTR2 staining percentage

| Subtype (Reference = Lactotroph) | Odds Ratio | Sig. | 95% Confidence Interval | |
| --- | --- | --- | --- | --- |
|  |  |  | Lower Bound | Upper Bound |
| Thyrotrophs | 18.664 | 0.000 | 5.524 | 63.058 |
| Corticotrophs | 2.503 | 0.013 | 1.218 | 5.147 |
| Somatotrophs | 6.348 | 0.000 | 2.908 | 13.855 |
| Gonadotrophs | 3.485 | 0.000 | 1.844 | 6.584 |
| Null | 1.046 | 0.912 | 0.468 | 2.337 |

**Supplemental Table 4.** Multiple regression analysis performed to determine the association between SSTR2 staining intensity with age, sex, tumour lineages and tumour size.

|  | Odds Ratio | Sig. | 95% Confidence Interval | |
| --- | --- | --- | --- | --- |
|  |  |  | Lower Bound | Upper Bound |
| Age | 0.985 | 0.143 | 0.966 | 1.005 |
| Sex | 0.974 | 0.937 | 0.508 | 1.869 |
| Micro | 1.647 | 0.254 | 0.699 | 3.879 |
| Macro | 0^a^ |  |  |  |
| Thyrotrophs | 85.966 | 0.000 | 15.034 | 491.559 |
| Corticotrophs | 5.507 | 0.001 | 2.002 | 15.149 |
| Somatotroph | 10.039 | 0.000 | 3.619 | 27.845 |
| Gonadotrophs | 5.362 | 0.002 | 1.822 | 15.778 |
| Null | 2.790 | 0.065 | 0.936 | 8.313 |
| Lactotroph | 0^a^ |  |  |  |

**Supplemental Table 5.** Multiple regression analysis performed to determine the association between SSTR2 staining percentage with age, sex, tumour lineage and tumour size.

|  | Odds Ratio | Sig. | 95% Confidence Interval | |
| --- | --- | --- | --- | --- |
|  |  |  | Lower Bound | Upper Bound |
| Age | 0.992 | 0.426 | 0.973 | 1.012 |
| Sex | 0.955 | 0.887 | 0.506 | 1.802 |
| Micro | 1.202 | 0.666 | 0.520 | 2.777 |
| Macro | 0^a^ |  |  |  |
| Thyrotrophs | 108.824 | 0.000 | 23.675 | 500.219 |
| Corticotrophs | 3.133 | 0.025 | 1.155 | 8.502 |
| Somatotroph | 17.742 | 0.000 | 6.343 | 49.629 |
| Gonadotrophs | 5.514 | 0.002 | 1.903 | 15.973 |
| Null | 2.772 | 0.064 | 0.944 | 8.140 |
| Lactotroph | 0^a^ |  |  |  |

**Supplemental Table 6.** Systematic Review Search Strategy and Terms.

| **Category** | **Terms** |
| --- | --- |
| **Primary Terms** | SSTR2 (Text word) |
|  | SSTR2A (Text word) |
|  | Somatostatin receptor 2 (Text word) |
| Pituitary Neuroendocrine Tumour Lineages | Growth Hormone- Secreting Pituitary Adenoma (MeSH Terms) |
|  | Prolactinoma (MeSH Terms) |
|  | ACTH- Secreting Pituitary Adenoma (MeSH Terms) |
|  | TSH- Secreting Adenomas (Text word) |
|  | TSH- Secreting Pituitary Adenoma (Text word) |
|  | Thyrotropin-Secreting Pituitary Tumours[Text Word] |
|  | Thyrotropin-Secreting Pituitary Adenomas[Text Word] |
|  | Thyrotroph Adenomas[Text Word] |
|  | Gonadotroph Adenoma[Text Word] |
|  | \| Functioning Gonadotroph Adenoma[Text Word] \| \| --- \| |
|  | \|  \| Gonadotroph Pituitary Adenoma[Text Word] \| \| --- \| --- \| |
|  | \|  \| FSH-Secreting Adenoma[Text Word] \| \| --- \| --- \| |
|  | \|  \| FSH-Secreting Pituitary Adenoma[Text Word] \| \| --- \| --- \| |
|  | \|  \| Follicle-Stimulating Hormone-Secreting Pituitary Adenoma[Text Word] \| \| --- \| --- \| |
|  | \|  \| LH-Secreting Adenoma[Text Word] \| \| --- \| --- \| |
|  | \|  \| LH-Secreting Pituitary Adenoma[Text Word] \| \| --- \| --- \| |
|  | \|  \| Luteinizing Hormone-Secreting Pituitary Adenoma[Text Word] \| \| --- \| --- \| |
|  | \|  \| Null Cell Adenoma[Text Word] \| \| --- \| --- \| |
|  | \|  \| Non-Functional Adenomas[Text Word] \| \| --- \| --- \| |
|  | \| Pituitary Neoplasm[Text Word] \| \| --- \| |
|  | \|  \| Pituitary Neuroendocrine Tumour[Text Word] \| \| --- \| --- \| |
|  | \|  \| PiNET[Text Word] \| \| --- \| --- \| |
| **Filters Applied** | Language: English[Filter] |
|  | Population: Humans[Filter] |
|  | Publication Date: 1990:2023[pdat] |
| **Search Logic** | Used AND and OR operators to combine terms effectively. |
|  | Specific subsets included for refining the search to relevant studies on pituitary adenomas. |

**Supplemental Table 7.** SSTR2 Expression by Pituitary Adenoma Lineage Comparison with Existing Literature.

| **Type of Tumour** | **Number of tumours with SSTR2 expression**  ***Present Study*** | **Number of tumours with SSTR2 expression**  ***Fuchs et al (50)*** | **Number of tumours with SSTR2 expression**  ***Remaining studies in systematic review*** |
| --- | --- | --- | --- |
| **Thyrotrophs** | 100% | 18% | 89-100%  [38, 51 52] |
| **Somatotrophs** | 95.3% | 64% | 72-100%  [21-35,37, 38] |
| **Gonadotrophs** | 82.8% | 15% | 0-100%  [38 , 46 ,47 ,58 ,64] |
| **Corticotrophs** | 75.9% | 28% | 0-100%  [38, 55 , 56] |
| **Lactotrophs** | 55.6% | 22% | 0-100%  [38 , 57] |
| **Null Cell adenomas** | 47.2% | 22% | 0-80%  [63, 66] |

**Supplemental Figures**

**Supplemental Figure 1.** SSTR2 Scoring (A) 0 - No staining (B) 1- Weak staining (C) 2- Moderate staining (D) 3- High staining.


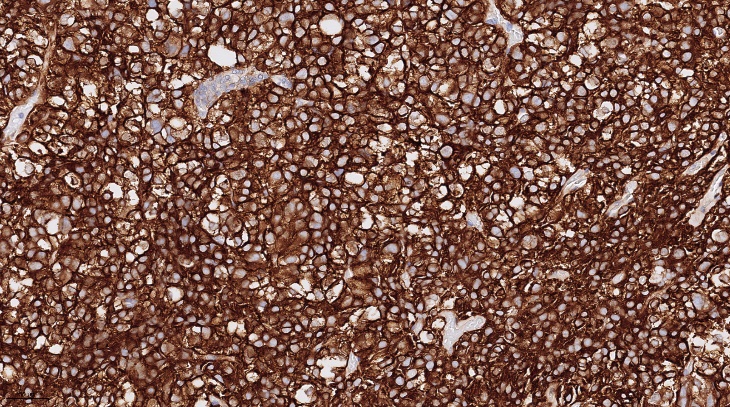

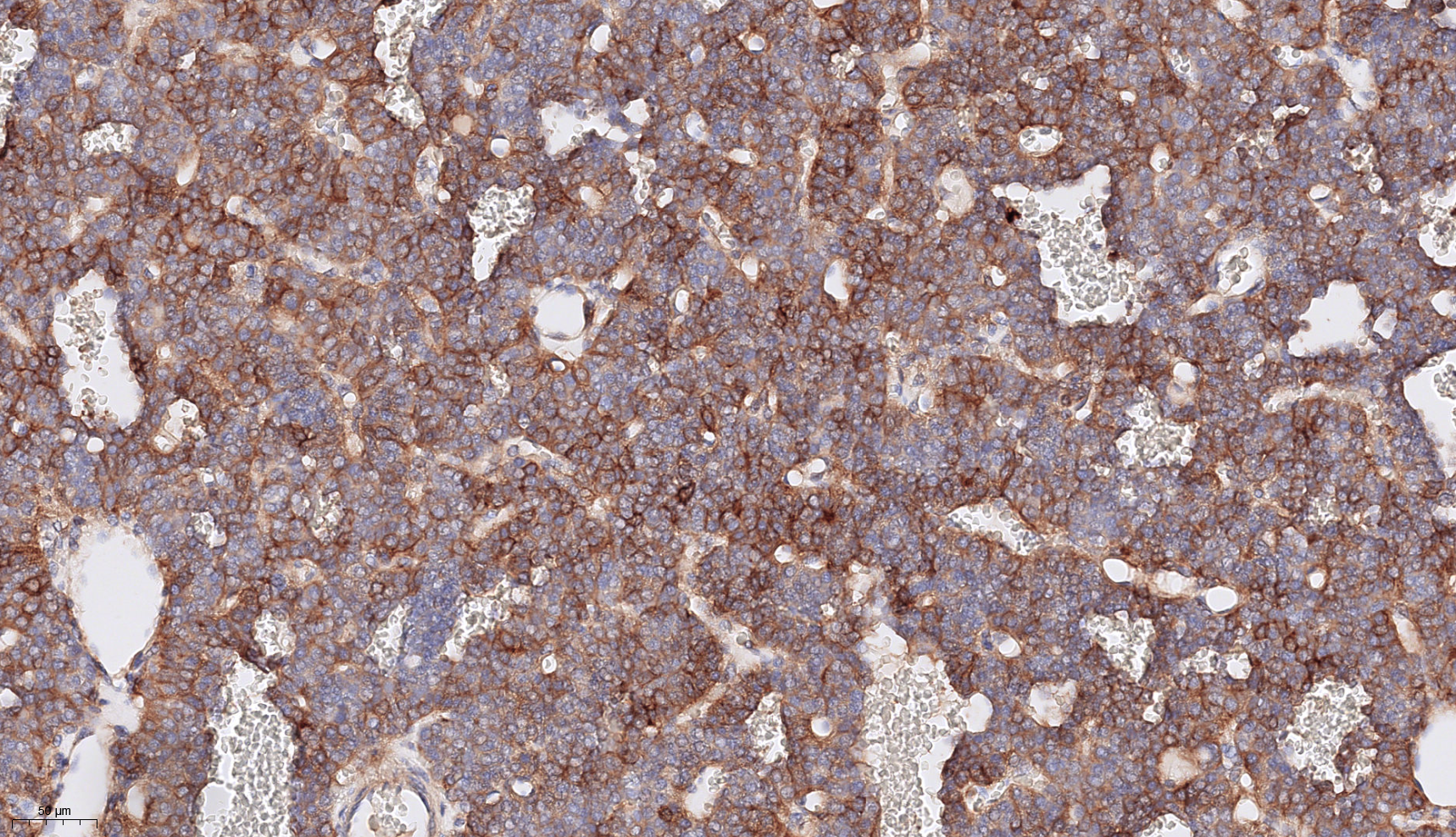

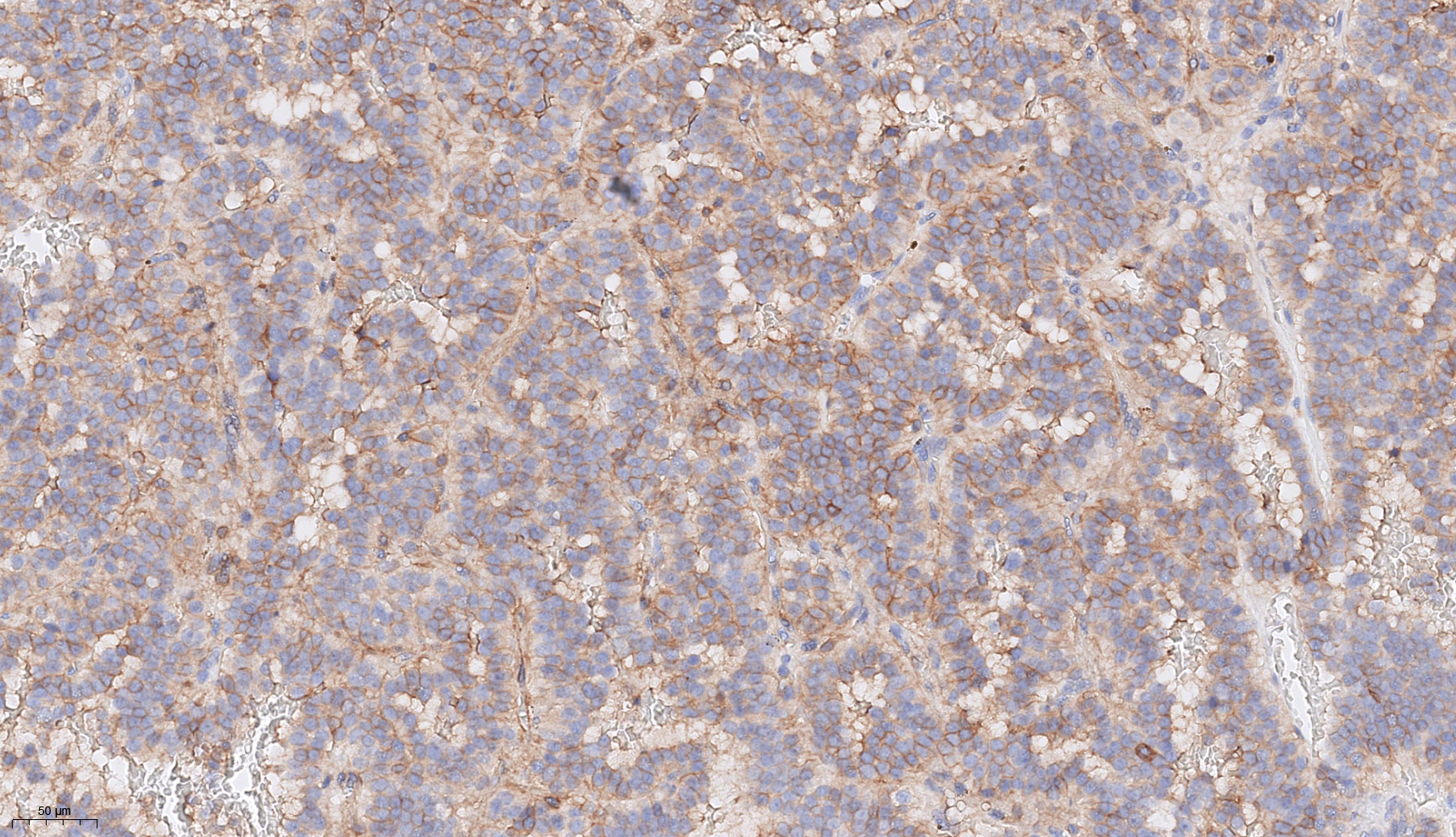

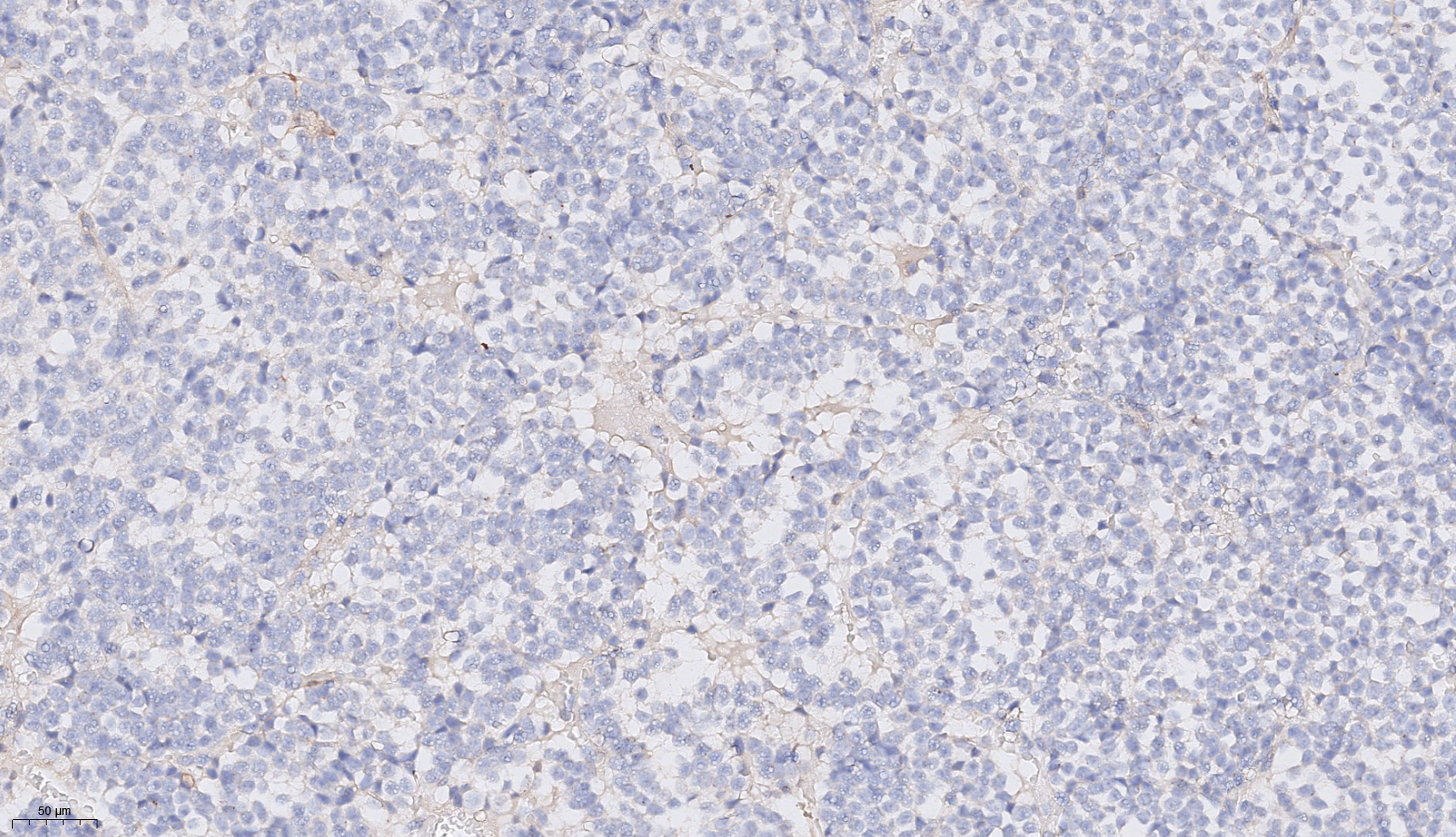


**A**

**B**

**C**

**D**

**Supplemental Figure 2.** PRISMA flow diagram of system review.

Records identified from*:

Databases (n = 149)

Records removed before screening:

Duplicate records removed (n = 0)

Records screened

(n = 149)

Records excluded**

Review Article (n = 9)

Retracted Article (n = 1)

Reports assessed for eligibility

(n = 139)

Reports excluded:

Beyond Scope

(n = 27)

Lack of access

(n = 11)

RNA expression only (n = 56)

Records identified from:

Citation searching

(n = 2)

Reports assessed for eligibility

(n = 2)

Reports excluded:

Beyond Scope

(n = 0)

RNA expression only (n = 1)

Studies included in review

(n = 46)

**Identification of studies via databases and registers**

**Identification of studies via other methods**

**Identification**

**Screening**

**Included**

**Supplemental References**

1. Y. Hu *et al.*, "Role of Somatostatin Receptor in Pancreatic Neuroendocrine Tumor Development, Diagnosis, and Therapy," (in eng), *Front Endocrinol (Lausanne),* vol. 12, p. 679000, 2021, doi: 10.3389/fendo.2021.679000.
2. M. Lechner *et al.*, "Somatostatin receptor 2 expression in nasopharyngeal cancer is induced by Epstein Barr virus infection: impact on prognosis, imaging and therapy," (in eng), *Nat Commun,* vol. 12, no. 1, p. 117, Jan 5 2021, doi: 10.1038/s41467-020-20308-8.
3. M. Lechner *et al.*, "Clinical outcomes, Kadish-INSICA staging and therapeutic targeting of somatostatin receptor 2 in olfactory neuroblastoma," *Eur J Cancer,* vol. 162, pp. 221-236, Feb 2022, doi: 10.1016/j.ejca.2021.09.046.
4. Detre S, Saclani Jotti G, Dowsett M. A “quickscore” method for immunohistochemical semiquantitation: validation for oestrogen receptor in breast carcinomas. *J Clin Pathol*. 1995;48(9):876–878.
5. X. Fan *et al.*, "Expression of somatostatin receptor subtype 2 in growth hormone-secreting pituitary adenoma and the regulation of miR-185," *Journal of Endocrinological Investigation,* vol. 38, no. 10, pp. 1117-1128, 2015/10/01 2015, doi: 10.1007/s40618-015-0306-7.
6. D. Iacovazzo *et al.*, "Factors predicting pasireotide responsiveness in somatotroph pituitary adenomas resistant to first-generation somatostatin analogues: an immunohistochemical study," (in English), *European Journal of Endocrinology,* vol. 174, no. 2, pp. 241-250, 01 Feb. 2016 2016, doi: 10.1530/eje-15-0832.
7. L. Kasuki, L. E. Wildemberg, L. V. Neto, J. Marcondes, C. M. Takiya, and M. R. Gadelha, "Ki-67 is a predictor of acromegaly control with octreotide LAR independent of SSTR2 status and relates to cytokeratin pattern," (in eng), *Eur J Endocrinol,* vol. 169, no. 2, pp. 217-23, Aug 2013, doi: 10.1530/eje-13-0349.
8. M. Takei *et al.*, "Immunohistochemical detection of somatostatin receptor (SSTR) subtypes 2A and 5 in pituitary adenoma from acromegalic patients: good correlation with preoperative response to octreotide," (in eng), *Endocr Pathol,* vol. 18, no. 4, pp. 208-16, Winter 2007, doi: 10.1007/s12022-007-9004-0.
9. O. Casar-Borota *et al.*, "Expression of SSTR2a, but not of SSTRs 1, 3, or 5 in somatotroph adenomas assessed by monoclonal antibodies was reduced by octreotide and correlated with the acute and long-term effects of octreotide," (in eng), *J Clin Endocrinol Metab,* vol. 98, no. 11, pp. E1730-9, Nov 2013, doi: 10.1210/jc.2013-2145.
10. L. E. Wildemberg *et al.*, "Low somatostatin receptor subtype 2, but not dopamine receptor subtype 2 expression predicts the lack of biochemical response of somatotropinomas to treatment with somatostatin analogs," (in eng), *J Endocrinol Invest,* vol. 36, no. 1, pp. 38-43, Jan 2013, doi: 10.3275/8305.
11. L. E. Wildemberg *et al.*, "Validation of immunohistochemistry for somatostatin receptor subtype 2A in human somatotropinomas: comparison between quantitative real time RT-PCR and immunohistochemistry," (in eng), *J Endocrinol Invest,* vol. 35, no. 6, pp. 580-4, Jun 2012, doi: 10.3275/7906.
12. U. Plöckinger *et al.*, "DG3173 (somatoprim), a unique somatostatin receptor subtypes 2-, 4- and 5-selective analogue, effectively reduces GH secretion in human GH-secreting pituitary adenomas even in Octreotide non-responsive tumours," (in eng), *Eur J Endocrinol,* vol. 166, no. 2, pp. 223-34, Feb 2012, doi: 10.1530/eje-11-0737.
13. L. Fougner, O. C. Borota, J. P. Berg, J. K. Hald, J. Ramm-Pettersen, and J. Bollerslev, "The clinical response to somatostatin analogues in acromegaly correlates to the somatostatin receptor subtype 2a protein expression of the adenoma," (in eng), *Clin Endocrinol (Oxf),* vol. 68, no. 3, pp. 458-65, Mar 2008, doi: 10.1111/j.1365-2265.2007.03065.x.
14. M. C. A. Coelho *et al.*, "Clinical significance of filamin A in patients with acromegaly and its association with somatostatin and dopamine receptor profiles," (in eng), *Sci Rep,* vol. 9, no. 1, p. 1122, Feb 4 2019, doi: 10.1038/s41598-018-37692-3.
15. L. Rass *et al.*, "Differences in somatostatin receptor subtype expression in patients with acromegaly: new directions for targeted therapy?," (in eng), *Hormones (Athens),* vol. 21, no. 1, pp. 79-89, Mar 2022, doi: 10.1007/s42000-021-00327-w.
16. M. Nakashima, K. Takano, and A. Matsuno, "Analyses of factors influencing the acute effect of octreotide in growth hormone-secreting adenomas," (in eng), *Endocr J,* vol. 56, no. 2, pp. 295-304, 2009, doi: 10.1507/endocrj.k08e-305.
17. L. Kasuki *et al.*, "AIP expression in sporadic somatotropinomas is a predictor of the response to octreotide LAR therapy independent of SSTR2 expression," (in eng), *Endocr Relat Cancer,* vol. 19, no. 3, pp. L25-9, Jun 2012, doi: 10.1530/erc-12-0020.
18. H. Pisarek, M. Pawlikowski, J. Kunert-Radek, and K. Winczyk, "Does the response of GH-secreting pituitary adenomas to octreotide depend on the cellular localization of the somatostatin receptor subtypes SSTR2 and SSTR5?," (in eng), *Endokrynol Pol,* vol. 61, no. 2, pp. 178-81, Mar-Apr 2010.
19. E. Thodou, G. Kontogeorgos, D. Theodossiou, and M. Pateraki, "Mapping of somatostatin receptor types in GH or/and PRL producing pituitary adenomas," (in eng), *J Clin Pathol,* vol. 59, no. 3, pp. 274-9, Mar 2006, doi: 10.1136/jcp.2005.026914.
20. C. G. B. Herkenhoff *et al.*, "Survivin: A Potential Marker of Resistance to Somatostatin Receptor Ligands," (in eng), *J Clin Endocrinol Metab,* vol. 108, no. 4, pp. 876-887, Mar 10 2023, doi: 10.1210/clinem/dgac610.
21. S. Chiloiro *et al.*, "Pasireotide and Pegvisomant Combination Treatment in Acromegaly Resistant to Second-Line Therapies: A Longitudinal Study," (in eng), *J Clin Endocrinol Metab,* vol. 104, no. 11, pp. 5478-5482, Nov 1 2019, doi: 10.1210/jc.2019-00825.
22. L. Chinezu *et al.*, "Expression of somatostatin receptors, SSTR2A and SSTR5, in 108 endocrine pituitary tumors using immunohistochemical detection with new specific monoclonal antibodies," (in eng), *Hum Pathol,* vol. 45, no. 1, pp. 71-7, Jan 2014, doi: 10.1016/j.humpath.2013.08.007.
23. J. Soukup *et al.*, "Predictive and prognostic significance of tumour subtype, SSTR1-5 and e-cadherin expression in a well-defined cohort of patients with acromegaly," (in eng), *J Cell Mol Med,* vol. 25, no. 5, pp. 2484-2492, Mar 2021, doi: 10.1111/jcmm.16173.
24. K. Kiseljak-Vassiliades *et al.*, "Differential somatostatin receptor (SSTR) 1-5 expression and downstream effectors in histologic subtypes of growth hormone pituitary tumors," (in eng), *Mol Cell Endocrinol,* vol. 417, pp. 73-83, Dec 5 2015, doi: 10.1016/j.mce.2015.09.016.
25. M. Kato *et al.*, "Differential expression of genes related to drug responsiveness between sparsely and densely granulated somatotroph adenomas," (in eng), *Endocr J,* vol. 59, no. 3, pp. 221-8, 2012, doi: 10.1507/endocrj.ej11-0177.
26. S. L. Fougner, O. Casar-Borota, A. Heck, J. P. Berg, and J. Bollerslev, "Adenoma granulation pattern correlates with clinical variables and effect of somatostatin analogue treatment in a large series of patients with acromegaly," (in eng), *Clin Endocrinol (Oxf),* vol. 76, no. 1, pp. 96-102, Jan 2012, doi: 10.1111/j.1365-2265.2011.04163.x.
27. B. Mayr, R. Buslei, M. Theodoropoulou, G. K. Stalla, M. Buchfelder, and C. Schöfl, "Molecular and functional properties of densely and sparsely granulated GH-producing pituitary adenomas," (in eng), *Eur J Endocrinol,* vol. 169, no. 4, pp. 391-400, Oct 2013, doi: 10.1530/eje-13-0134.
28. D. Cuevas-Ramos *et al.*, "A structural and functional acromegaly classification," (in eng), *J Clin Endocrinol Metab,* vol. 100, no. 1, pp. 122-31, Jan 2015, doi: 10.1210/jc.2014-2468.
29. S. E. Franck *et al.*, "Somatostatin Receptor Expression in GH-Secreting Pituitary Adenomas Treated with Long-Acting Somatostatin Analogues in Combination with Pegvisomant," (in eng), *Neuroendocrinology,* vol. 105, no. 1, pp. 44-53, 2017, doi: 10.1159/000448429.
30. A. Muhammad *et al.*, "Pasireotide Responsiveness in Acromegaly Is Mainly Driven by Somatostatin Receptor Subtype 2 Expression," (in eng), *J Clin Endocrinol Metab,* vol. 104, no. 3, pp. 915-924, Mar 1 2019, doi: 10.1210/jc.2018-01524.
31. U. Plöckinger *et al.*, "Selective loss of somatostatin receptor 2 in octreotide-resistant growth hormone-secreting adenomas," (in eng), *J Clin Endocrinol Metab,* vol. 93, no. 4, pp. 1203-10, Apr 2008, doi: 10.1210/jc.2007-1986.
32. F. Gatto *et al.*, "In Vitro Head-to-Head Comparison Between Octreotide and Pasireotide in GH-Secreting Pituitary Adenomas," (in eng), *J Clin Endocrinol Metab,* vol. 102, no. 6, pp. 2009-2018, Jun 1 2017, doi: 10.1210/jc.2017-00135.
33. E. Venegas-Moreno *et al.*, "Association between dopamine and somatostatin receptor expression and pharmacological response to somatostatin analogues in acromegaly," (in eng), *J Cell Mol Med,* vol. 22, no. 3, pp. 1640-1649, Mar 2018, doi: 10.1111/jcmm.13440.
34. T. L. Fuchs, L. Sioson, A. Sheen, A. Clarkson, and A. J. Gill, "Immunohistochemical expression of somatostatin receptors SSTR2A and SSTR5 in 299 pituitary adenomas," (in eng), *Pathology,* vol. 50, no. 4, pp. 472-474, Jun 2018, doi: 10.1016/j.pathol.2017.10.024.
35. E. Thodou and G. Kontogeorgos, "Somatostatin receptor profile in pituitary thyrotroph adenomas," (in eng), *Clin Neurol Neurosurg,* vol. 195, p. 105865, Aug 2020, doi: 10.1016/j.clineuro.2020.105865.
36. E. L. Wang *et al.*, "Clinicopathological characterization of TSH-producing adenomas: special reference to TSH-immunoreactive but clinically non-functioning adenomas," (in eng), *Endocr Pathol,* vol. 20, no. 4, pp. 209-20, Winter 2009, doi: 10.1007/s12022-009-9094-y.
37. H. J. Fang *et al.*, "Short-term Preoperative Octreotide for Thyrotropin-secreting Pituitary Adenoma," (in eng), *Chin Med J (Engl),* vol. 130, no. 8, pp. 936-942, Apr 20 2017, doi: 10.4103/0366-6999.204098.
38. N. G. Herguido *et al.*, "Surgical Outcome and Treatment of Thyrotropin-Secreting Pituitary Tumors in a Tertiary Referral Center," (in eng), *World Neurosurg,* vol. 130, pp. e634-e639, Oct 2019, doi: 10.1016/j.wneu.2019.06.180.
39. R. van der Pas *et al.*, "Preoperative normalization of cortisol levels in Cushing's disease after medical treatment: consequences for somatostatin and dopamine receptor subtype expression and in vitro response to somatostatin analogs and dopamine agonists," (in eng), *J Clin Endocrinol Metab,* vol. 98, no. 12, pp. E1880-90, Dec 2013, doi: 10.1210/jc.2013-1987.
40. J. A. Veit *et al.*, "Detection of paranasal ectopic adrenocorticotropic hormone-secreting pituitary adenoma by Ga-68-DOTANOC positron-emission tomography-computed tomography," (in eng), *Laryngoscope,* vol. 123, no. 5, pp. 1132-5, May 2013, doi: 10.1002/lary.23867.
41. H. Lasolle, A. Vasiljevic, F. Borson-Chazot, and G. Raverot, "Pasireotide: A potential therapeutic alternative for resistant prolactinoma," (in eng), *Ann Endocrinol (Paris),* vol. 80, no. 2, pp. 84-88, Apr 2019, doi: 10.1016/j.ando.2018.07.013.
42. M. Lee *et al.*, "SSTR3 is a putative target for the medical treatment of gonadotroph adenomas of the pituitary," (in eng), *Endocr Relat Cancer,* vol. 22, no. 1, pp. 111-9, Feb 2015, doi: 10.1530/erc-14-0472.
43. A. Fusco *et al.*, "Treatment with octreotide LAR in clinically non-functioning pituitary adenoma: results from a case-control study," (in eng), *Pituitary,* vol. 15, no. 4, pp. 571-8, Dec 2012, doi: 10.1007/s11102-011-0370-8.
44. C. Ramírez *et al.*, "Expression of Ki-67, PTTG1, FGFR4, and SSTR 2, 3, and 5 in nonfunctioning pituitary adenomas: a high throughput TMA, immunohistochemical study," (in eng), *J Clin Endocrinol Metab,* vol. 97, no. 5, pp. 1745-51, May 2012, doi: 10.1210/jc.2011-3163.
45. H. Pisarek, J. Kunert-Radek, M. Radek, J. Swietoslawski, K. Winczyk, and M. Pawlikowski, "Expression of somatostatin receptor subtypes in primary and recurrent gonadotropinomas: are somatostatin receptors involved in pituitary adenoma recurrence?," (in eng), *Neuro Endocrinol Lett,* vol. 32, no. 1, pp. 96-101, 2011.
46. A. Gruszka *et al.*, "The effect of selective sst1, sst2, sst5 somatostatin receptors agonists, a somatostatin/dopamine (SST/DA) chimera and bromocriptine on the "clinically non-functioning" pituitary adenomas in vitro," (in eng), *Life Sci,* vol. 78, no. 7, pp. 689-93, Jan 11 2006, doi: 10.1016/j.lfs.2005.05.061.
47. K. A. Øystese, O. Casar-Borota, K. R. Normann, M. Zucknick, J. P. Berg, and J. Bollerslev, "Estrogen Receptor α, a Sex-Dependent Predictor of Aggressiveness in Nonfunctioning Pituitary Adenomas: SSTR and Sex Hormone Receptor Distribution in NFPA," (in eng), *J Clin Endocrinol Metab,* vol. 102, no. 9, pp. 3581-3590, Sep 1 2017, doi: 10.1210/jc.2017-00792.
48. A. Tjörnstrand *et al.*, "Lower (68) Ga-DOTATOC uptake in nonfunctioning pituitary neuroendocrine tumours compared to normal pituitary gland-A proof-of-concept study," (in eng), *Clin Endocrinol (Oxf),* vol. 92, no. 3, pp. 222-231, Mar 2020, doi: 10.1111/cen.14144.
49. L. Chinezu, A. Vasiljevic, J. Trouillas, M. Lapoirie, E. Jouanneau, and G. Raverot, "Silent somatotroph tumour revisited from a study of 80 patients with and without acromegaly and a review of the literature," (in eng), *Eur J Endocrinol,* vol. 176, no. 2, pp. 195-201, Feb 2017, doi: 10.1530/eje-16-0738.
50. M. Pawlikowski, H. Pisarek, J. Kunert-Radek, and A. Radek, "Immunohistochemical detection of somatostatin receptor subtypes in "clinically nonfunctioning" pituitary adenomas," (in eng), *Endocr Pathol,* vol. 14, no. 3, pp. 231-8, Fall 2003, doi: 10.1007/s12022-003-0015-1.
51. J. H. He *et al.*, "SSTR2 is a prognostic factor and a promising therapeutic target in glioma," (in eng), *Am J Transl Res,* vol. 13, no. 10, pp. 11223-11234, 2021.
52. S. E. Franck *et al.*, "Somatostatin receptor expression in GH-secreting pituitary adenomas treated with long-acting somatostatin analogues in combination with pegvisomant," *Neuroendocrinology,* vol. 105, no. 1, pp. 44-53, 2017.
